# *Lactiplantibacillus plantarum* Fermentation Alters the Physicochemical Characteristics and Enhances the Antioxidant and Immunomodulatory Activities of *Eucommia ulmoides* Leaf Polysaccharides

**DOI:** 10.3390/foods15193463

**Published:** 2026-09-28

**Authors:** Bingyan Wang, Han Qiao, Chungang Han, Xuelei Zhang, Kang Cheng, Xiaoxiao Liang, Ke Wang, Yong Zhang

**Affiliations:** College of Bioengineering, Henan University of Technology, Zhengzhou 450001, China; 2024930718@stu.haut.edu.cn (B.W.); qiaohan137@126.com (H.Q.); 231060500304@stu.haut.edu.cn (C.H.); xueleizhang@haut.edu.cn (X.Z.); chengkang68@126.com (K.C.); lxx2024@huat.edu.cn (X.L.); kewang@haut.edu.cn (K.W.)

**Keywords:** solid-state fermentation, *Eucommia ulmoides* leaf polysaccharides, *Lactiplantibacillus plantarum*, antioxidant activity, immunomodulation, gut microbiota

## Abstract

*Eucommia ulmoides* leaf polysaccharides (EUPSs) are plant-derived macromolecules with antioxidant and immunomodulatory potential. This study investigated whether solid-state fermentation using *Lactiplantibacillus plantarum* modifies the physicochemical characteristics and bioactivity of EUPS. Unfermented and fermented polysaccharide fractions (EUPS and FEUPS, respectively) were compared for yield, monosaccharide composition, molecular weight, infrared characteristics, morphology, and radical-scavenging activity, and were further evaluated in cyclophosphamide-induced immunosuppressed mice. Fermentation increased the recovery yield of the dried polysaccharide fraction to 19.45%, representing a 55.1% increase compared with the unfermented extraction, and altered the monosaccharide composition and molecular weight distribution while largely preserving the major functional groups. FEUPS exhibited a looser, more porous morphology, and stronger DPPH•, ABTS•^+^, and hydroxyl radical-scavenging activities than EUPS. In vivo, FEUPS improved body weight gain, histopathological features, serum immunoglobulin levels, hepatic antioxidant status, and splenic gene-expression profiles. Compared with the corresponding EUPS groups, FEUPS showed significant differences in selected immune- and antioxidant-related parameters, particularly at the medium dose. FEUPS was also associated with changes in cecal microbiota composition and correlations between bacterial taxa and host immune and antioxidant markers. Overall, fermentation improved the yield and functional properties of EUPS, highlighting the potential of FEUPS for incorporation into functional food products.

## 1. Introduction

*Eucommia ulmoides* Oliv. is a relict plant from the Quaternary glacial period and has a long history of use in traditional Chinese medicine because of its abundant bioactive compounds. In 2023, the National Health Commission officially included *E. ulmoides* leaf in the “Medicinal and Edible Substances” catalog, opening up new opportunities to promote the value-added utilization of *E. ulmoides* leaf and its use in functional foods [1]. Among these, *E. ulmoides* leaf polysaccharides (EUPSs)—one of the primary active components—are macromolecular substances formed by the linkage of various monosaccharides via glycosidic bonds and possess a relatively complex structure [2]. Modern studies have demonstrated that EUPSs exhibit multiple biological activities, including immune-enhancing, antioxidant [3,4,5], anti-inflammatory [6,7,8,9,10], and hepatoprotective effects. Our previous research found that EUPS exerts antioxidant protection in the liver through enhanced SOD, CAT, and GSH-Px activities, elevated T-AOC, and reduced MDA levels, further demonstrating the potential value of EUPS as a natural functional ingredient [11]. However, improving the physicochemical characteristics and bioactivity of EUPS is still a major challenge for its further development and use.

Fermentation-based polysaccharide extraction is a food-grade processing strategy that can increase the yield of plant-derived polysaccharides and enhance their functional properties [12]. It utilizes enzyme systems produced by microorganisms during their growth and metabolism to degrade cell walls, thereby facilitating polysaccharide release. At the same time, microbial metabolites can also interact with polysaccharides, altering the monosaccharide profile, molecular weight distribution, and microstructure of the polysaccharide components, thereby affecting their biological activity [13]. Among these, *Lactiplantibacillus plantarum* is a common food-grade lactic acid bacterium that has been widely used in food fermentation processes and the development of probiotic products. Wang et al. [14] used *L. plantarum* CICC 23121-assisted fermentation to extract polysaccharides from Schisandra sphenanthera fruit, achieving a polysaccharide extraction rate of 11.12%, which was 53.38% higher than that obtained by hot-water extraction. Huang et al. [15] screened and isolated *L. plantarum* JGS49 from asparagus kimchi and confirmed that fermentation using this strain markedly enhanced the antioxidant activity of asparagus juice. These studies suggest that *L. plantarum* fermentation may be an effective method for improving polysaccharide extraction yields and bioactivity.

Previous studies have examined the effects of microbial fermentation on *E. ulmoides* products, including fermented tea and crude extracts, while *L. plantarum* fermentation has also been investigated in other plant-derived materials. However, direct evidence regarding how *L. plantarum* fermentation alters the polysaccharide fraction recovered specifically from *E. ulmoides* leaves remains limited. Therefore, this study used *L. plantarum* to ferment *E. ulmoides* leaves to investigate structural changes caused by fermentation and bioactivity of EUPS. By comparing the in vitro properties of EUPS and fermented *E. ulmoides* leaf polysaccharides (FEUPSs) and evaluating their immunoregulatory effects in a mouse model of cyclophosphamide-induced immunosuppression, the study also examined the relationship between bacterial communities and host immune and antioxidant markers. This study aimed to determine whether food-grade fermentation may enhance the physicochemical characteristics and bioactivity of EUPS and to lay the groundwork for the potential incorporation of FEUPS into functional food products.

## 2. Materials and Methods

### 2.1. Materials and Reagents

*E. ulmoides* leaves were collected in July from trees more than five years old and supplied by Henan Jinduzhong Agricultural Technology Co., Ltd. (Xuchang, China), *L. plantarum* was provided by the Animal Nutrition Laboratory of the School of Biological Engineering at Henan University of Technology (Zhengzhou, China). Cyclophosphamide (purity ≥ 98%) and levamisole hydrochloride (purity ≥ 99%) were both purchased from Shanghai Aladdin Biochemical Technology Co., Ltd. (Shanghai, CHina); PBS was sourced from Wuhan Savier Biotechnology Co., Ltd., Wuhan, China. Unless otherwise specified, all chemicals and reagents were of analytical grade. Unless otherwise specified, all reagents used in this section were purchased from Tianjin Kemiou Chemical Reagent Co., Ltd. (Tianjin, China).

### 2.2. Preparation of EUPS and FEUPS

*E. ulmoides* leaves were washed, dried, and ground, then sieved through a 60-mesh screen to obtain a uniform powder [11]. Based on fermentation conditions previously optimized in our laboratory, *E. ulmoides* leaf powder was mixed with an *L. plantarum* suspension (6 × 10^8^ CFU/mL) at an inoculum level of 1.0% (*v*/*w*) and a liquid-to-solid ratio of 0.6 mL/g, followed by static solid-state fermentation at 37 °C for 37 h. Upon completion of fermentation, based on our previous study [11], we applied ultrasound-assisted extraction to EUPS and FEUPS under the same conditions to improve polysaccharide recovery: distilled water was incorporated at 22 mL per gram of material, followed by ultrasonic extraction at 63 °C for 1 h. The extract was purified by ethanol precipitation and activated-carbon decolorization, followed by dialysis using a 3.5 kDa molecular weight cut-off membrane for 96 h and freeze-drying to obtain FEUPS. EUPS was prepared under the same incubation, extraction, and purification conditions as FEUPS, except that *L. plantarum* was not added. The polysaccharide extraction yield was calculated as the percentage of the dry polysaccharide fraction relative to the initial dry weight of the *E. ulmoides* leaf powder. The total polysaccharide content in the FEUPS and EUPS was quantified by the phenol–sulfuric acid method. Quantification was performed at 480 nm against a glucose standard curve (Y = 4.66x + 0.0664, R^2^ = 0.999).

### 2.3. Determination of the In Vitro Antioxidant Activity of FEUPS and EUPS

For each antioxidant assay, EUPS and FEUPS were prepared in distilled water at the same concentration series within the corresponding test range. For the DPPH• assay, 2 mL of sample solution was mixed with 2 mL of 0.1 mmol/L DPPH• solution and incubated in the dark at room temperature for 30 min before measurement at 517 nm, following the method of Fu et al. [16] with minor modifications. For the ABTS•^+^ assay, the working solution was prepared from 7 mmol/L ABTS and 2.45 mmol/L potassium persulfate and incubated in the dark for 16 h. After dilution to an absorbance of 0.70 ± 0.02 at 734 nm, 0.1 mL of sample solution was mixed with 6 mL of ABTS•^+^ working solution and incubated in the dark for 10 min before measurement at 734 nm. Hydroxyl radical-scavenging activity was determined using FeSO_4_, salicylic acid, and H_2_O_2_, with incubation at 37 °C for 1 h and measurement at 510 nm. Appropriate assay blanks and reagent controls were included; no external antioxidant reference standard was used. Each assay was performed in three independent analytical replicates using the same EUPS or FEUPS preparation [11].

### 2.4. Monosaccharide Composition Analysis

EUPS and FEUPS samples were hydrolyzed under identical conditions. The actual sample masses were 2.50 mg for FEUPS and 2.16 mg for EUPS. Each sample was hydrolyzed with 1 mL of 2 M TFA (Shanghai Aladdin Biochemical Technology Co., Ltd., Shanghai, China) at 121 °C for 2 h, followed by identical post-hydrolysis processing and a 20-fold dilution before HPAEC-PAD analysis. Monosaccharide composition was analyzed on an ICS-5000 system (Thermo Fisher Scientific, Waltham, MA, USA) fitted with a CarboPac PA20 column (3 × 150 mm; Thermo Fisher Scientific), using NaOH with the mobile phase delivered at 0.5 mL/min and 25 μL of sample injected.

### 2.5. Molecular Weight Determination of E. ulmoides Polysaccharides

The molecular weight profile was obtained using a PL-GPC 50 system with refractive index detection (Agilent Technologies, Santa Clara, CA, USA). A 0.1 M NaNO_3_ solution flowed through the column at 1.0 mL/min. Samples (20 μL) were injected while the column was maintained at 30 °C. Dextran standards of different molecular weights were used to establish a calibration curve between retention time and log molecular weight, which was used to calculate the molecular weight parameters of EUPS and FEUPS. The original GPC chromatograms and molecular-weight calibration profiles are shown in Figure S1.

### 2.6. Structural and Morphological Characterization by FT-IR and SEM

FT-IR was used to analyze the functional group structure of polysaccharides: an appropriate amount of dried sample was ground to a uniform powder and combined with dried potassium bromide (KBr), then pressed into transparent pellets. Spectral measurements were obtained with a Nicolet iS20 FT-IR spectrometer (Thermo Fisher Scientific, Waltham, MA, USA) in the 4000–400 cm^−1^ range. Each spectrum represented 32 scans collected at 4 cm^−1^ resolution, and a pure KBr pellet served as the background reference.

Scanning electron micrographs were obtained with a Gemini SEM 300 microscope (Carl Zeiss Microscopy GmbH, Jena, Germany) operated at 5 kV. Micrographs were obtained at magnifications of 5000× and 10,000×.

### 2.7. Animal Experiment Design

This study used 54 healthy male BALB/c mice (18.0 ± 2 g), purchased from Henan Scibes Biotechnology Co, Zhengzhou, China (Animal Production License No.: SCXK (Yu) 2020-0005). All animal experiments followed the animal-use procedures approved by the Institutional Animal Care and Use Committee of Henan University of Technology (HAUTETHI2025-0923).

Animals were maintained under controlled conditions of 25 ± 0.5 °C and 55 ± 2% relative humidity with a 12 h light–dark cycle and free access to food and water. After a one-week acclimation period, mice were randomly allocated into nine experimental groups using a random-number method (*n* = 6 biological replicates per group): the normal control group (CON), the model group (MOD), the low-dose FEUPS group (FEL), the medium-dose FEUPS group (FEM), the high-dose FEUPS group (FEH), the low-dose EUPS group (EL), the medium-dose EUPS group (EM), the high-dose EUPS group (EH), and the levamisole hydrochloride group (LEV) as a positive control group. Mice in each group were housed in three cages, with two mice per cage, and the individual mouse was considered the experimental unit.

Starting on day 8, an immunosuppression model was established and maintained for 3 days. Mice in the CON group were administered an equivalent volume of saline intraperitoneally, whereas those in the remaining groups received cyclophosphamide (CTX) intraperitoneally at 80 mg/kg. Following model establishment, the CON and MOD groups received saline by oral gavage once daily. Based on previous studies on the biological activities of natural polysaccharides, the FEUPS and EUPS groups received the corresponding polysaccharides by oral gavage at doses of 100, 200, and 400 mg/kg, respectively, with the administered amounts adjusted according to the measured total polysaccharide content of each preparation [17]. The LEV group received levamisole at 40 mg/kg by oral gavage. Each group underwent continuous treatment on days 11–24. Following completion of the experimental period, food was withheld from the mice for 12 h without water restriction; they were then euthanized, and samples were collected for subsequent parameter analysis. Because different treatment groups required different gavage solutions, blinding was not applied during the administration period. After sample collection, samples were coded before subsequent biochemical, molecular, and microbiota analyses to minimize potential assessment bias.

### 2.8. Body Weight Changes and Organ Indices

Body weight was recorded every 3 days, and body weight gain was calculated. After euthanasia, the heart, liver, spleen, and kidneys were excised, rinsed with pre-chilled PBS, blotted dry, and weighed.

$$Organ\;index\;\left(\%\right)=Organ\;weight\;\left(g\right)/Body\;weight\;\left(g\right)\times 100\%$$

### 2.9. Histopathological Examination

Tissue samples obtained from the spleens and livers of mice were immersed in 4% paraformaldehyde for 24 h. Tissue samples underwent graded ethanol dehydration and paraffin embedding before being cut into 4 μm sections and stained with H&E. Tissue changes were observed, and images were captured for analysis. Representative sections were selected from the entire tissue area and photographed at the same magnification. Histopathological changes in the liver and spleen were qualitatively assessed based on tissue architecture, hepatocyte arrangement, and the morphology of the splenic white pulp; no quantitative scoring system for lesion severity was used.

### 2.10. Determination of Immunoglobulin Levels in Mouse Serum

Following blood collection, serum was obtained by allowing the blood to clot for 1 h at room temperature, followed by centrifugation at 3500 rpm for 10 min. The levels of IgG and IgM in the serum were determined using an ELISA kit (Ruixin Biotechnology Co., Ltd., Quanzhou, China), as specified in the kit instructions.

### 2.11. Assessment of Antioxidant Capacity in Mouse Liver

Antioxidant-associated indices, including superoxide dismutase (SOD) and catalase (CAT) activities, reduced glutathione (GSH) and malondialdehyde (MDA) contents, and total antioxidant capacity (T-AOC), were determined using the respective commercial assay kits (Boxbio Science and Technology Co., Ltd., Beijing, China). Liver tissues were homogenized with ice-cold physiological saline at a tissue-to-saline ratio of 1:9 (*w*/*v*). The experimental procedures for each assay followed the instructions supplied with the corresponding kit.

### 2.12. Determination of Spleen-Related Gene Expression

Mouse spleen tissue was collected and promptly frozen in liquid nitrogen, followed by storage at −80 °C. Total RNA was extracted from the spleen tissue according to the manufacturer’s instructions of the RNA extraction kit (Vazyme Biotech Co., Ltd., Nanjing, China). RNA yield and purity were determined with a NanoDrop 2000 spectrophotometer (Thermo Fisher Scientific, Waltham, MA, USA). The extracted RNA was subsequently converted into cDNA using a reverse transcription kit (Vazyme Biotech Co., Ltd., Nanjing, China). The resulting cDNA was used as the template to quantify the expression of spleen immune-related genes by real-time quantitative PCR (RT-qPCR) with a qTOWER3G real-time PCR system (Analytik Jena AG, Jena, Germany). The amplification program consisted of an initial denaturation at 95 °C for 30 s, followed by 40 cycles of 95 °C for 10 s and 60 °C for 30 s. Melting-curve analysis was performed after amplification to assess amplification specificity. β-actin served as the internal reference gene for normalization. Relative gene expression was calculated using the 2^−ΔΔCt^ method and presented as fold change. Primer sequences are listed in Table S1.

### 2.13. Cecal Microbiome Analysis

Cecal samples were processed by Shanghai Meiji Biotechnology Co., Ltd. (Shanghai, China) for microbial 16S rRNA gene sequencing. Total DNA was obtained using the E.Z.N.A.^®^ Soil DNA Kit (Omega Bio-Tek, Norcross, GA, USA), with its quality and quantity assessed through 1.0% agarose gel electrophoresis and NanoDrop 2000 spectrophotometry (Thermo Scientific, Waltham, MA, USA). The bacterial 16S rRNA gene was amplified using the primers 338F (5′–ACTCCTACGGGAGGCAGCAG–3′) and 806R (5′–GGACTACHVGGGTWTCTAAT–3′), followed by purification (YuHua kit, Shanghai, China) and Qubit 4.0 quantification (Thermo Fisher Scientific, Waltham, MA, USA). Sequencing was performed on the Illumina NextSeq 2000 system (Illumina, Inc., San Diego, CA, USA). Sequence reads were first subjected to quality control with fastp (v0.23.4), followed by paired-end read merging using FLASH (v1.2.11). Sequences were clustered into operational taxonomic units (OTUs) at 97% sequence similarity using USEARCH (v11), followed by chimera removal. Taxonomic classification was performed using the RDP Classifier (v2.11) against the SILVA 138.2/16s_bacteria database with a confidence threshold of 70%. Samples were rarefied to 36,737 sequences per sample, and 885 OTUs were retained for downstream analyses. Alpha diversity, PCoA, NMDS, LEfSe, and Spearman’s correlation analyses were performed on the Majorbio cloud platform. Differences in overall microbial community structure were assessed using PERMANOVA based on Bray–Curtis distances, and multivariate dispersion was evaluated using PERMDISP.

### 2.14. Data Processing and Statistical Analysis

Raw experimental data were organized and preprocessed in Microsoft Excel. One-way ANOVA was performed using SAS 9.2 statistical software, and graphs were plotted using GraphPad Prism 11.0.2. All data are presented as mean ± SEM. Normality was assessed using the Shapiro–Wilk test and homogeneity of variance was evaluated using Levene’s test. When both assumptions were satisfied, differences among groups were analyzed using one-way ANOVA followed by Tukey’s post hoc test. When data were approximately normally distributed, but homogeneity of variance was not satisfied, Welch’s ANOVA followed by the Games–Howell post hoc test was used. For data that did not satisfy the normality assumption, the Kruskal–Wallis test followed by Dunn’s multiple-comparison test was applied. A value of *p* < 0.05 was considered statistically significant. For multiple Spearman correlation tests between bacterial taxa and host parameters, *p* values were adjusted using the Benjamini–Hochberg false discovery rate (FDR) procedure, with an FDR-adjusted *p* value (q value) < 0.05 considered statistically significant. A summary of the statistical methods applied to the different experimental datasets is provided in Table S2.

## 3. Results

### 3.1. Antioxidant Activity and Characterization of FEUPS and EUPS

The recovery yield of the dried FEUPS fraction reached 19.45%, representing a 55.1% increase compared with that of EUPS (Figure 1A, *p* < 0.05). As shown in Figure 1B–D, EUPS and FEUPS exhibited different radical-scavenging activities at the tested concentrations. As the concentration increased from 0.3 to 2 mg/mL, the DPPH• radical-scavenging activity of FEUPS showed an upward trend, reaching a maximum of 95.23% at 2 mg/mL; EUPS reached its highest scavenging rate of 75.39% at 1.5 mg/mL (Figure 1B). In the ABTS•^+^ assay (Figure 1C), as the sample concentration increased from 0.1 to 0.5 mg/mL, both polysaccharides exhibited a gradual increase in radical-scavenging activity, with FEUPS exhibiting higher activity than EUPS throughout the tested concentration range. Similarly, in the hydroxyl radical scavenging assay (Figure 1D), the scavenging activities generally increased over the concentration range of 10–30 mg/mL, with FEUPS consistently exhibiting higher activity than EUPS. Overall, FEUPS exhibited significantly stronger in vitro free radical-scavenging activity than EUPS (*p* < 0.05).

As shown in Table 1, 10 types of monosaccharides were detected in both EUPS and FEUPS, with arabinose (Ara) and galactose (Gal) as the major monosaccharides. Their relative proportions increased from 27.91% and 36.98% in EUPS to 31.14% and 43.77% in FEUPS, respectively, whereas the relative proportion of Glc decreased from 17.15% to 7.64%.

As shown in Figure 2A,B, EUPS and FEUPS exhibited different molecular weight distribution profiles. EUPS showed a relatively narrow molecular weight distribution, with the main peak at approximately 28 kDa, a low-molecular-weight fraction in the 10–20 kDa range, and a weak high-molecular-weight tail. After fermentation, the main peak of FEUPS shifted to approximately 37 kDa, the low-molecular-weight fraction in the 10–20 kDa range decreased, and the tail in the high-molecular-weight region (>80 kDa) became more pronounced. The detailed molecular weight distribution parameters are presented in Table S3. Fermentation resulted in a small increase in PDI (1.55–1.60), accompanied by greater molecular weight heterogeneity. FT-IR analysis of EUPS and FEUPS (Figure 2C,D) showed distinct absorption bands at 3430 cm^−1^ (O–H stretching vibration), 2921 cm^−1^ (C–H stretching vibration), 1637 cm^−1^ (H–O–H bending vibration of bound water), 1572 cm^−1^ and 1384 cm^−1^ (COO^−^ stretching vibrations), and 1743 cm^−1^ (C=O stretching vibration of ester carbonyl groups), indicating that the major characteristic functional groups were largely retained after fermentation. Microscopic morphological observations (Figure 2E–H) revealed that the surface morphology of FEUPS underwent marked changes. EUPS showed a compact, smooth surface morphology, whereas fermentation produced a markedly more porous and irregular structure in FEUPS, with granular and lamellar features.

### 3.2. Effects of FEUPS and EUPS on Body Weight Changes and Organ Indices in Mice

Changes in body weight and body weight gain are shown in Figure 3A,B. Body weight increased progressively in the CON group over the period from day 10 to day 24. In contrast to the CON group, the MOD group displayed lower body weight gain, whereas the LEV, FEL, FEM, and EM groups showed higher body weight gain than the MOD group (Figure 3A). Figure 3B shows a significant reduction in body weight gain in the MOD group relative to the CON group (*p* < 0.05). Mice in the LEV, FEL, FEM, and EM groups exhibited significantly higher body weight gain relative to the MOD group (*p* < 0.05), reaching levels comparable to that of the CON group. As shown in Table 2, numerical differences in organ indices were observed among the experimental groups, but these differences were not statistically significant.

### 3.3. Effects of FEUPS and EUPS on the Liver and Spleen

As shown in Figure 4A, the CON group exhibited a well-preserved splenic architecture, with clearly distinguishable red and white pulp and densely arranged lymphocytes. In the MOD group, the spleen tissue showed white pulp atrophy, blurred boundaries between splenic regions, and reduced lymphocyte density. Relative to MOD mice, each treatment group showed varying degrees of improvement. Among them, the FEM group exhibited a clearer white pulp structure and a denser distribution of lymphocytes; the spleen tissue morphology in the LEV group more closely resembled that observed in the CON group. As shown in Figure 4B, mice in the CON group exhibited well-preserved liver architecture, with hepatocytes arranged in an orderly manner and normal cell morphology. In the MOD group, hepatocytes were disorganized, with loose cytoplasm and focal inflammatory cell infiltration. Similarly, relative to MOD mice, the LEV and FEM groups displayed more regular liver architecture and less pronounced vacuolization.

### 3.4. Effects of FEUPS and EUPS on Serum Immunoglobulins and Liver Antioxidant Status

Significantly lower serum IgG and IgM concentrations were detected in the MOD group than in controls (Figure 5A,B). The MOD group also showed lower hepatic CAT and SOD activities and GSH and T-AOC levels, together with a higher MDA level (*p* < 0.05). Following intervention, these indicators exhibited different levels of improvement. No significant differences were detected between the FEM and LEV groups for the measured serum immunoglobulin and hepatic antioxidant indices (*p* > 0.05). Compared with the corresponding EUPS groups, the FEUPS groups, particularly the FEL and FEM groups, demonstrated a significant increase in IgM, SOD activity, and T-AOC (*p* < 0.05). In addition, MDA levels were lower following FEUPS intervention. Overall, FEUPS showed significant differences from EUPS in selected serum immunoglobulin and hepatic antioxidant parameters, particularly at the low and medium doses.

### 3.5. Effects of FEUPS and EUPS on the Expression Levels of Spleen-Related Genes

The transcriptional responses of selected genes were evaluated using RT-qPCR (Figure 6). Relative to the CON group, the MOD group showed significantly reduced expression of T-cell-related genes (*Cd3e*, *Cd4*, *Cd8a*, and *Cd18*), immunoregulatory factors (*Ifng*, *IL-2*, and *IL-10*), and *Nrf2*, accompanied by significant upregulation of *Bax* and *IL-6* (*p* < 0.05). Following polysaccharide intervention, the expression levels of most of the aforementioned genes approached the levels observed in the CON group to varying degrees. Notably, among the polysaccharide groups, the FEM group showed relatively pronounced regulatory effects across multiple measured genes. Except for *Cd8a*, no significant differences were detected between the FEM and LEV groups in the expression levels of the measured genes (*p* > 0.05). Compared with the corresponding EM group, the FEM group exhibited significant increases in these genes (*Cd3e*, *Cd4*, *Cd8a*, *Ifng*, *IL-2*, *IL-10*, and *Nrf2*), accompanied by a marked reduction in *IL-6* expression (*p* < 0.05), whereas *Cd18* and *Bax* expression remained statistically unchanged. Detailed fold-change values for the expression of the analyzed genes are provided in Table S4.

### 3.6. Effects of FEUPS and EUPS on the Gut Microbiota of Mice Under Immune Stress

The α-diversity analysis (Figure 7A–D) showed no significant differences in the ACE, Chao1, or Shannon indices among groups (*p* > 0.05), whereas the Simpson index differed significantly among groups (*p* < 0.05). PCoA and NMDS showed partial separation in the spatial distribution of samples among the experimental groups (Figure 7E,F; NMDS stress = 0.231). PERMANOVA based on Bray–Curtis distances confirmed a significant overall difference in microbial community structure among the groups (R^2^ = 0.193, F = 1.349, *p* = 0.008), whereas PERMDISP showed no significant difference in multivariate dispersion among the groups (F = 1.411, *p* = 0.222).

Community composition analysis is shown in Figure 8A,B. At the phylum level (Figure 8A), Bacillota and Bacteroidota predominated, together comprising over 80% of the total abundance. Relative to the CON group, the Bacillota abundance decreased while that of Bacteroidota increased in the MOD group. Following polysaccharide intervention, the relative abundance of Bacillota increased to different extents in all groups, especially the FEM and FEH groups, bringing the F/B ratio closer to that of the CON group. Detailed phylum-level relative abundance data are provided in Table S5. At the genus level (Figure 8B), the microbial community was dominated by norank_f__Muribaculaceae, Lachnospiraceae_NK4A136_group, *Desulfovibrio*, and *Lactobacillus*. Lachnospiraceae_NK4A136_group showed a pronounced decrease in relative abundance in the MOD group compared with the CON group. Following FEUPS intervention, this genus showed increased relative abundance and a dose-related trend. The LEV group exhibited the most pronounced changes in microbial community composition, with a further decline in genera associated with the Lachnospiraceae family and the appearance of additional microbial taxa. Detailed genus-level relative abundance data are provided in Table S6. As shown in Figure 8C, heatmap analysis revealed distinct gut microbial profiles across the experimental groups. In the MOD group, the relative abundances of beneficial bacterial genera, such as *Ligilactobacillus*, Lachnospiraceae_NK4A136_group, *Roseburia*, and *Blautia*, were reduced; conversely, the relative proportions of the potentially pathogenic genera *Desulfovibrio* and *Helicobacter* were increased. Following intervention, the medium- and high-dose groups of FEUPS and EUPS partially reversed the aforementioned trends. Sample cluster analysis showed that the MOD group formed an independent cluster and displayed a microbial profile that differed from the control group; in contrast, some treatment groups showed partial shifts in microbial community composition toward profiles more similar to the CON group; however, complete clustering with the CON group was not observed.

LEfSe analysis (LDA score ≥ 2) was used to further screen for discriminative taxa across the groups. The LEfSe cladogram showed distinct taxonomic enrichment patterns among the groups, including differential enrichment of lineages within Lachnospiraceae, Muribaculaceae, and Lactobacillaceae (Figure 9A). As shown in Figure 9B, the CON group was primarily enriched in [Eubacterium]_siraeum_group and NK4A214_group, while the MOD group was primarily enriched in Butyricicoccaceae, *Agathobacter*, and *Helicobacter*. Each intervention group exhibited distinct biomarker profiles: *Bifidobacterium* was enriched in the FEL group, Christensenellaceae in the FEH group, *Ligilactobacillus* in the EL group, and *Anaerostipes* in the EH group. No significantly enriched taxa were identified in the FEM group. Based on the analysis of community composition, the microbial profile of the FEM group showed greater similarity to the CON group. Associations among these parameters were further evaluated using Spearman correlation analysis (Figure 9C). After Benjamini–Hochberg FDR correction, several bacterial taxa remained significantly associated with host immune and antioxidant parameters. In particular, several taxa, including the [Eubacterium] siraeum group, *Colidextribacter*, *Alistipes*, *Roseburia*, *Ligilactobacillus*, *Ruminococcus*, the Lachnospiraceae_NK4A136_group, norank_f__Muribaculaceae, and unclassified Lachnospiraceae, showed positive associations with immune- and antioxidant-related parameters, whereas *Desulfovibrio* and *Helicobacter* generally showed opposite association patterns. Significance levels shown in Figure 9 are based on FDR-adjusted *p* values.

## 4. Discussion

The antioxidant properties of EUPS were substantially strengthened after *L. plantarum fermentation*, consistent with the observations of Liu et al. [18] in fermented *E. ulmoides* tea. This stronger activity may be associated with alterations in the composition and physicochemical characteristics of the polysaccharides during fermentation [19]. Specifically, microbial enzymes may degrade the plant cell wall matrix and disrupt interactions, including hydrogen bonds linking polysaccharides with other cell wall constituents, thereby exposing more reducing ends and hydrogen-donating groups that may contribute to free radical scavenging [20]. The different concentration-response patterns observed in the DPPH• and ABTS•^+^ assays may reflect differences in the reaction characteristics of the two radical systems and the concentration ranges tested. In addition, antioxidant activity was expressed as radical-scavenging percentage rather than Trolox equivalents, which may limit direct quantitative comparison with studies using standardized antioxidant-equivalent measurements. Tian et al. [21] reported that fermentation mediated by *Bacillus* sp. DU-106 alters the molecular weight of FDP-1, supporting the ability of microbial fermentation to modify polysaccharide characteristics. Earlier research has suggested that Ara-rich polysaccharides may possess greater antioxidant and immunomodulatory potential [22,23]. In this study, fermentation altered the monosaccharide composition of EUPS, and the main molecular weight peak shifted toward the high-molecular-weight region [24]. The increased proportion of higher-molecular-weight fractions after fermentation, together with the largely preserved major functional groups, suggests that this change may be associated with an increased degree of polymerization in some polysaccharide fractions rather than extensive chemical structural reconstruction or changes in monosaccharide types. SEM observations revealed a morphological transition from a dense, smooth, block-like structure to a loose, porous, granular structure. These structural changes may increase the accessible surface area of the polysaccharides, potentially improving their interactions with water and biological systems and thereby enhancing their biological activity [25]. The improved antioxidant performance of FEUPS was accompanied by changes in monosaccharide composition, molecular weight distribution, and surface morphology. Based on previous studies, these physicochemical changes may contribute to differences in radical-scavenging activity. In addition, because the purity of the polysaccharide preparations was not fully characterized and microbial extracellular polysaccharides were not separately isolated or quantified, the observed differences cannot be attributed exclusively to fermentation-induced modification of plant-derived polysaccharides.

Changes in body weight and organ indices often serve as general indicators for evaluating the physiological and immune status of mice. CTX treatment significantly reduced body weight gain, whereas only numerical differences in organ indices were observed among groups, without reaching statistical significance. The liver is crucial for xenobiotic metabolism and detoxification [26]. Damage to liver structure is typically accompanied by severe oxidative stress and metabolic disorders [27,28,29]. The spleen is a major secondary lymphoid organ involved in T- and B-cell activation and immune responses [30,31]. CTX administration was accompanied by splenic white-pulp atrophy and disruption of the normal arrangement of hepatocytes [32,33,34]. FEUPS treatment increased body weight gain, alleviated splenic atrophy, and improved white pulp morphology. These changes suggest that during CTX-induced immunosuppression, FEUPS may protect splenic structure and improve immune function. FEUPS also alleviated liver tissue damage. This protective effect may be related to its stronger antioxidant activity.

Humoral immunity is crucial for the host defense system. Its function is often evaluated by measuring serum immunoglobulin levels. Significant decreases in serum IgG and IgM levels were observed following CTX treatment, which is consistent with impaired humoral immunity [35]. After polysaccharide treatment, IgG and IgM levels increased to different degrees. The FEM group showed values close to those of the LEV group of the immune and antioxidant parameters measured. These results indicate that FEUPS may reduce the humoral immunosuppression caused by CTX. Similarly, previous studies have reported that fermented clam polysaccharides can modulate immunoglobulin-related indices and improve the morphology of immune organs [36]. As an important organ involved in CTX metabolism, the liver is susceptible to oxidative damage induced by its active metabolites [37]. CAT and SOD are key antioxidant enzymes responsible for scavenging hydrogen peroxide and superoxide anions, while GSH, as an important non-enzymatic antioxidant, participates in the reduction of peroxides and the scavenging of free radicals [38,39,40]. T-AOC provides an overall measure of enzymatic and non-enzymatic antioxidant capacity, while MDA is a typical biomarker of lipid peroxidation [41,42,43]. In the present study, compared with the corresponding doses of EUPS, FEUPS showed more pronounced improvements in several antioxidant parameters, particularly at the low and medium doses, as evidenced by significantly increased SOD activity and T-AOC levels, together with lower MDA levels. These findings suggest that fermentation may further enhance the in vivo antioxidant potential of EUPS. This pattern is consistent with the stronger free radical-scavenging activity of FEUPS observed in the in vitro assays, further supporting the view that fermentation may enhance the antioxidant activity of the resulting polysaccharide fraction. Notably, no significant differences were detected between the FEM and LEV groups for multiple immune and antioxidant indicators (*p* > 0.05). Furthermore, the antioxidant parameters did not show a consistent increase with increasing dose, and the medium-dose FEUPS group exhibited a more consistent improvement across the measured indices, suggesting that the antioxidant response may not follow a simple linear dose-dependent relationship. Overall, these findings indicate that FEUPS alleviated CTX-induced oxidative imbalance and showed greater effects than unfermented EUPS for selected antioxidant parameters.

The immunomodulatory effects of natural polysaccharides in the spleen may involve the regulation of T-cell-related genes and cytokines [43]. *Cd3e*, *Cd4*, *Cd8a*, and *Cd18* are all key genes involved in T-cell immune responses. Among these, *Cd3e* is involved in T-cell activation signal transduction; *Cd4* and *Cd8a* serve as surface markers for helper T cells and cytotoxic T cells, respectively, and are involved in antigen recognition; and *Cd18* mediates leukocyte adhesion and migration [44,45,46]. Evidence from previous research indicates that polysaccharides from Mesona chinensis Benth. exhibit immunomodulatory activity by regulating T lymphocytes [47]. Furthermore, similar regulatory effects have also been reported for other fermented polysaccharides [48,49]. *Nrf2* is an important regulator of cellular redox homeostasis, while *Bax* is closely associated with apoptotic damage under immunosuppressive stress [50,51]. *Ifng* and *IL-2* are closely related to cell-mediated immune responses and T-cell activation, whereas *IL-10* and *IL-6* are involved in the regulation of inflammatory responses [52,53]. In this study, FEUPS treatment was associated with changes in the splenic mRNA expression of genes related to T-cell markers, cytokines, antioxidant responses, and apoptosis. These transcriptional changes indicate associations with immune- and oxidative-stress-related processes. Following FEUPS intervention, the expression levels of these genes generally shifted toward those observed in the normal control group, as reflected by increased expression of T-cell-related genes, immunoregulatory factors, and *Nrf2*, together with reduced *IL-6* expression. These findings suggest that FEUPS may alleviate CTX-induced immunosuppression by reducing oxidative and inflammatory stress, suppressing abnormal apoptosis-related responses, and maintaining T-cell-associated immune function. However, because the present study assessed only the mRNA expression of these genes and did not further examine the protein levels or activation states of *Bax*, *IL-6*, or related signaling proteins, these relationships should be regarded as a hypothetical model based on transcriptional changes rather than a confirmed molecular mechanism.

The fermentation process can reshape the molecular weight profile and surface morphology of polysaccharides through microbial enzymes and metabolic activities. Such changes may affect how gut microorganisms utilize polysaccharides. The gut microbiota is crucial for host immune homeostasis and redox balance [51,54]. Evidence from earlier studies suggests that polysaccharides can reshape the gut microbial community by altering the relative abundance of particular bacterial taxa [55,56,57]. In this study, changes in the physicochemical characteristics of EUPS resulting from fermentation may affect the extent to which they are utilized by gut microbiota. These structural changes may also provide more favorable conditions for the growth and metabolism of certain bacterial populations. The changes in gut microbial composition observed following FEUPS intervention may be associated with these fermentation-induced physicochemical changes. Several taxa identified by LEfSe may be biologically relevant to the observed host responses. For example, members of Lachnospiraceae and genera such as *Roseburia* have frequently been associated with intestinal microbial metabolism, whereas *Helicobacter* has been linked to intestinal inflammatory conditions [58]. *Ligilactobacillus* represents another host-associated bacterial group that may respond to dietary interventions [59]. Correlation analyses also indicate that changes in gut microbial composition are associated with immune-related genes, serum immunoglobulins, and hepatic antioxidant markers. No significantly enriched taxa were identified in the FEM group by LEfSe. The community composition analyses suggested that the overall microbial profile of the FEM group was more similar to that of the CON group than that of some other treatment groups. While these correlation analyses indicate an association between changes in gut microbial composition, host immune function, and antioxidant status, they do not prove a direct causal relationship between the two. Further studies combining short-chain fatty acid analysis and antibiotic intervention are required to further investigate the contribution of the gut microbiota to the immunomodulatory effects of FEUPS.

## 5. Conclusions

The present study demonstrated that solid-state fermentation with *Lactiplantibacillus plantarum* altered the physicochemical characteristics of the polysaccharide fraction recovered from *E. ulmoides* leaves and enhanced its functional activity. Fermentation also increased the recovery yield of the dried polysaccharide fraction by 55.1% compared with the unfermented extraction. Compared with unfermented EUPS, FEUPS exhibited stronger in vitro antioxidant activity, together with changes in monosaccharide composition and molecular weight profile and a more open, porous surface structure. In vivo, FEUPS was associated with changes in body weight gain, histological features, serum immunoglobulin levels, hepatic antioxidant indices, splenic gene expression, and cecal microbiota composition in CTX-treated mice. These findings support the antioxidant and immunomodulatory potential of the FEUPS preparation but do not establish specific signaling pathways, lymphocyte functional changes, or microbiota-mediated mechanisms. In addition, because the purity of the polysaccharide preparations was not fully characterized and microbial extracellular polysaccharides were not separately quantified, the observed effects cannot be attributed exclusively to plant-derived polysaccharides modified by fermentation. Further protein-level, cellular, and mechanistic studies are required.

## Figures and Tables

**Figure 1:**
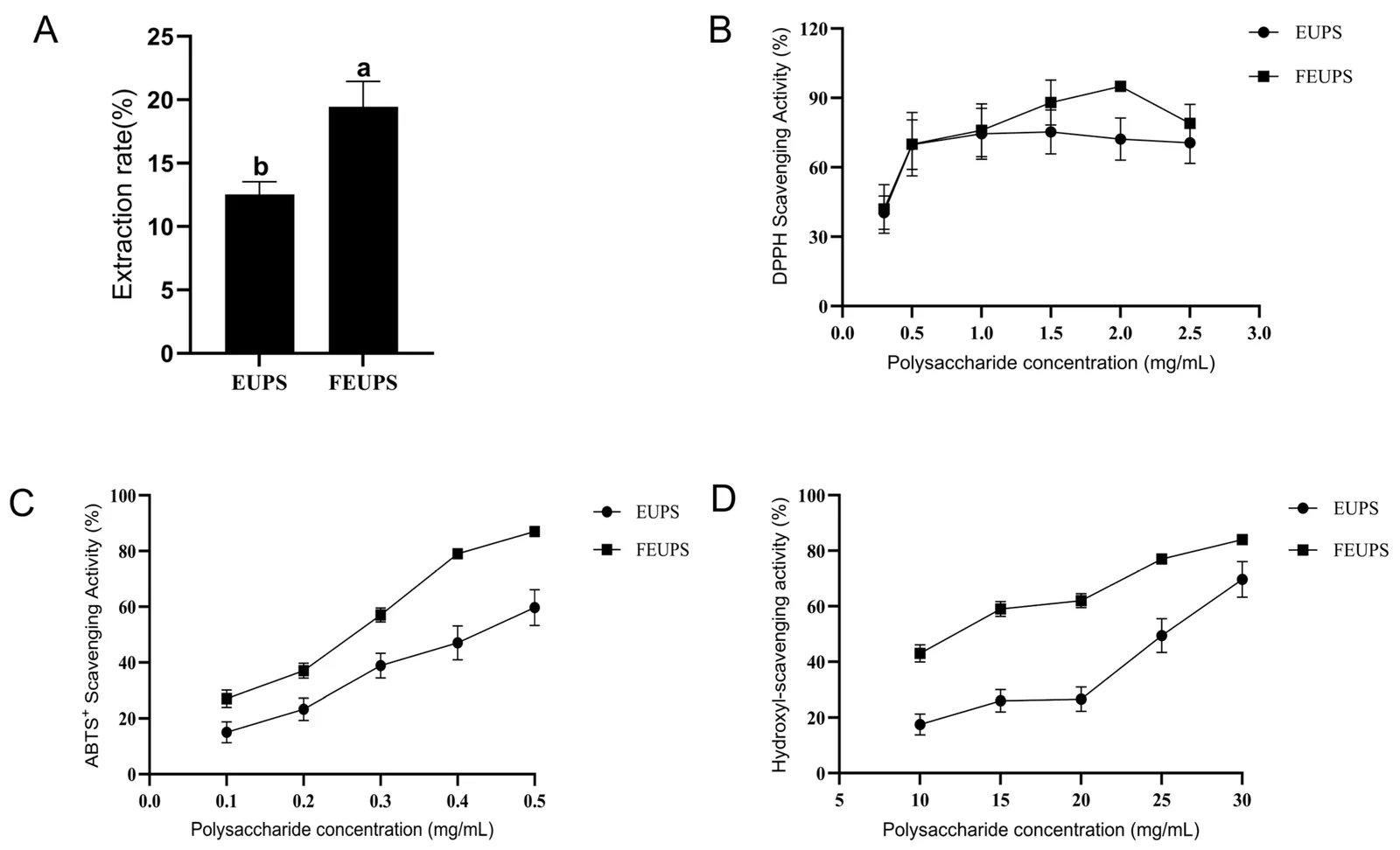
Effects of *L. plantarum* fermentation on the yield and antioxidant activity of EUPS and FEUPS. (**A**) Changes in the extraction yield of polysaccharides; (**B**–**D**) radical scavenging activities: (**B**) DPPH•, (**C**) ABTS•^+^, and (**D**) hydroxyl radical. In the free radical scavenging experiment, the data (a,b) are presented as mean ± SEM (*n* = 3).

**Figure 2:**
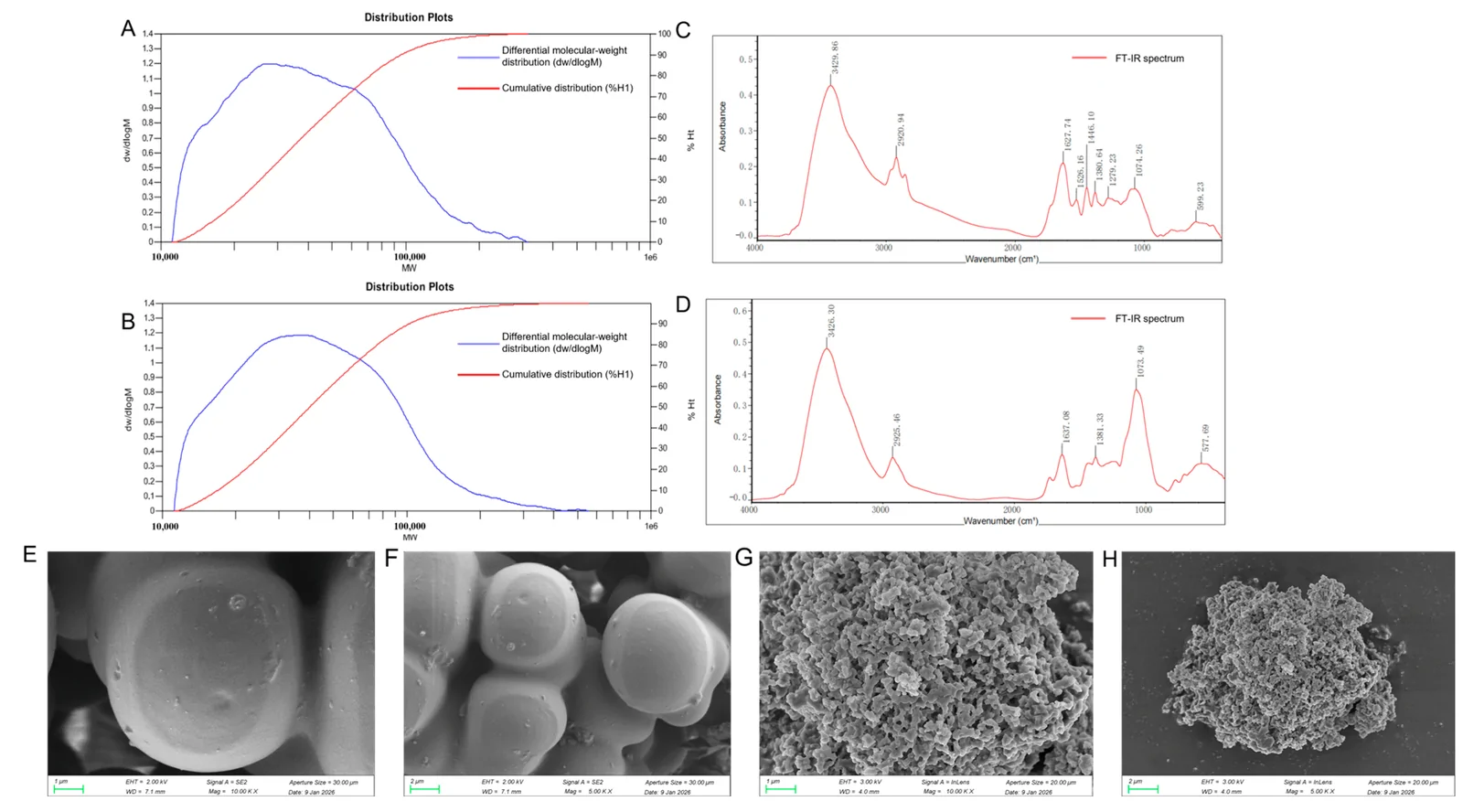
Effects of *L. plantarum* fermentation on the physicochemical characteristics and surface morphology of EUPS and FEUPS. (**A**,**B**) Molecular weight distribution of EUPS and FEUPS; (**C**,**D**) FT-IR spectra of EUPS and FEUPS; In (**A**,**C**), the blue lines represent the differential molecular-weight distribution (dw/dlogM), and the red lines represent the cumulative distribution (%H1). In (**B**,**D**), the red lines represent the FT-IR absorbance spectra. In (**B**,**D**), the red lines represent the FT-IR absorbance spectra. (**E**–**H**) SEM images of EUPS—(**E**) 10.0 k×, (**F**) 5.0 k×—and FEUPS—(**G**) 10.0 k×, (**H**) 5.0 k×.

**Figure 3:**
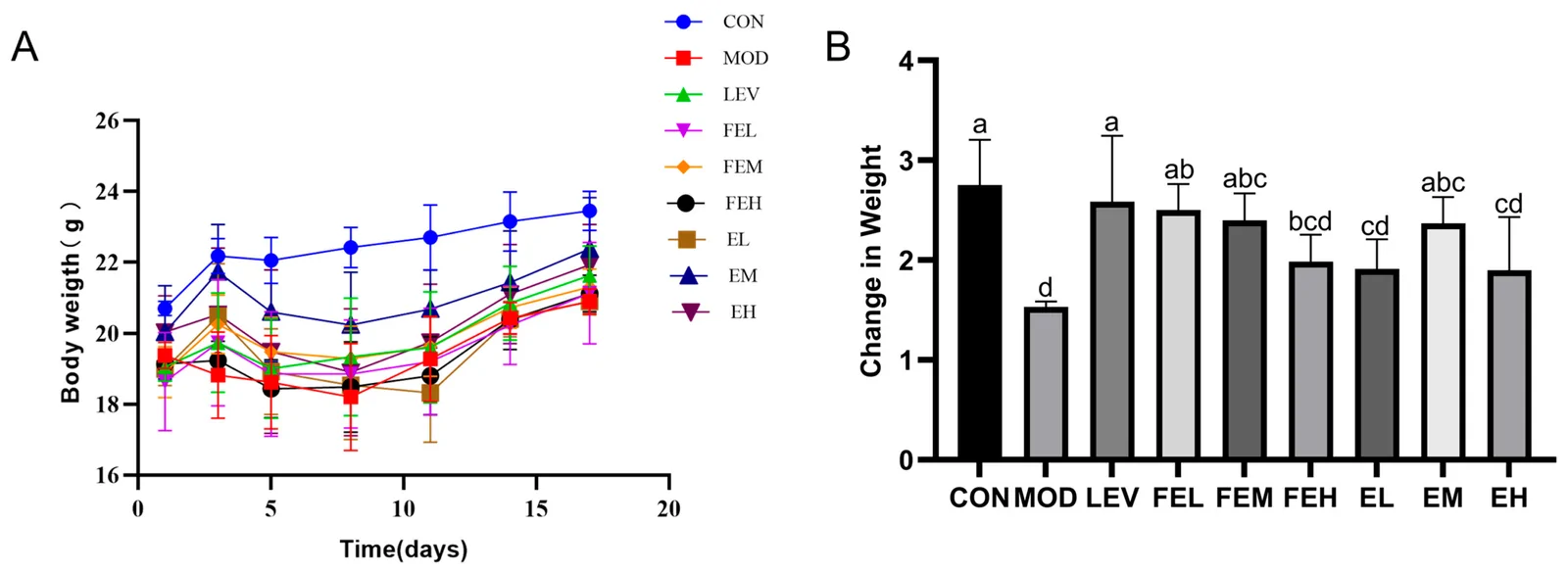
Effects of FEUPS and EUPS on body weight in CTX-induced immunosuppressed mice. (**A**) Changes in body weight during the experimental period; (**B**) body weight gain. Data are presented as mean ± SEM (*n* = 6). Different lowercase letters indicate significant differences among groups at *p* < 0.05. Serial body-weight measurements were used to illustrate temporal changes, whereas statistical comparisons among groups were performed on body weight gain over the experimental period.

**Figure 4:**
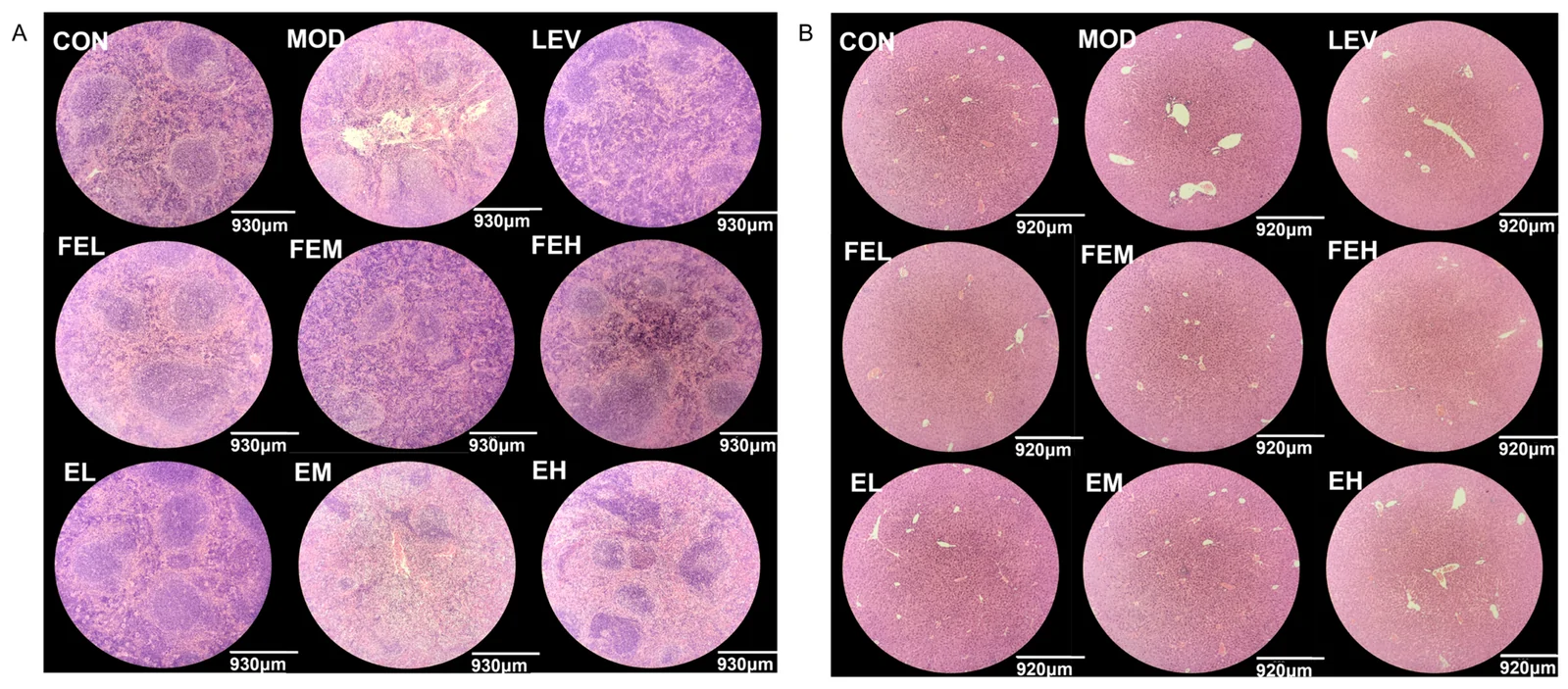
Effects of FEUPS and EUPS on the histopathological morphology of the spleen and liver in cyclophosphamide-induced immunosuppressed mice. (**A**) Representative H&E-stained sections of spleen tissue; (**B**) representative H&E-stained sections of liver tissue. Six biological replicates were examined per group (*n* = 6).

**Figure 5:**
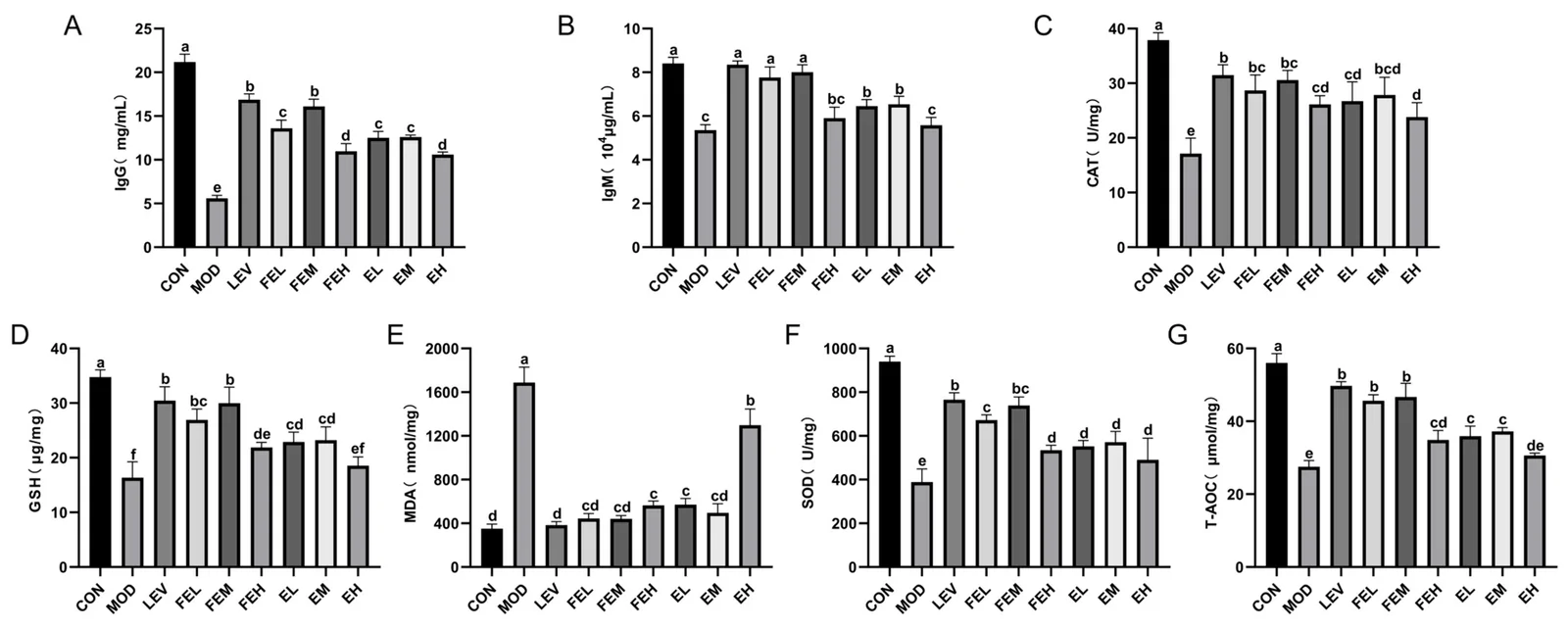
Effects of FEUPS and EUPS on serum immunoglobulins and liver antioxidant status. Serum levels of (**A**) IgG and (**B**) IgM; hepatic antioxidant indices, including (**C**) CAT activity, (**D**) GSH level, (**E**) MDA level, (**F**) SOD activity, and (**G**) T-AOC. Data are presented as the mean ± standard error (*n* = 6). Different lowercase letters indicate significant differences among groups at *p* < 0.05.

**Figure 6:**
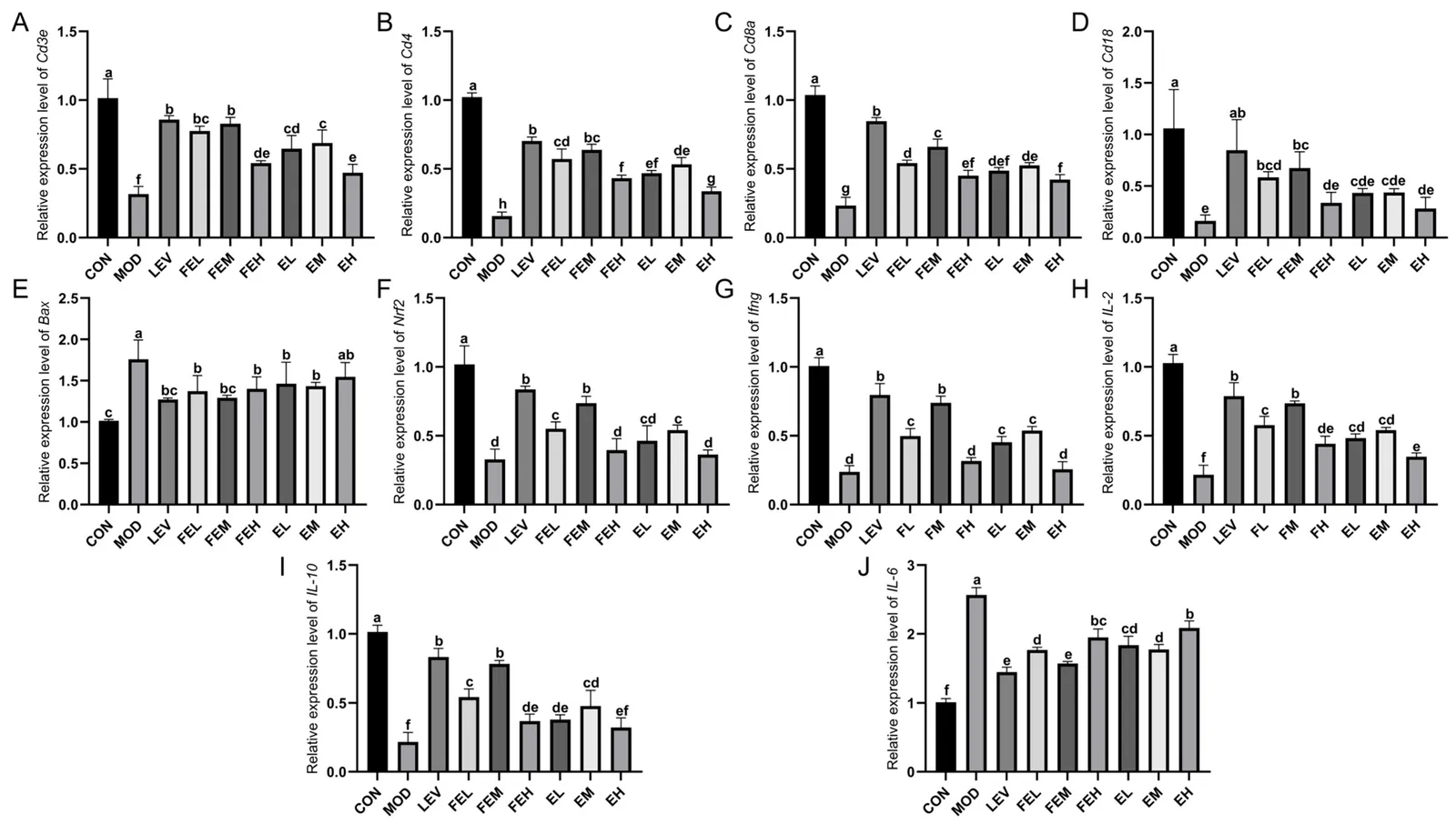
Effects of FEUPS and EUPS on the expression levels of spleen-related genes. T-cell-related genes: (**A**) *Cd3e*; (**B**) *Cd4*; (**C**) *Cd8a*; (**D**) *Cd18*. Oxidative stress-related gene expression: (**E**) *Bax*; (**F**) *Nrf2*. Inflammatory cytokine-related gene expression: (**G**) *Ifng*; (**H**) *IL-2*; (**I**) *IL-10*; (**J**) *IL-6*. Data are presented as mean ± SEM (*n* = 6). Different letters indicate statistically significant differences at the *p* < 0.05 level.

**Figure 7:**
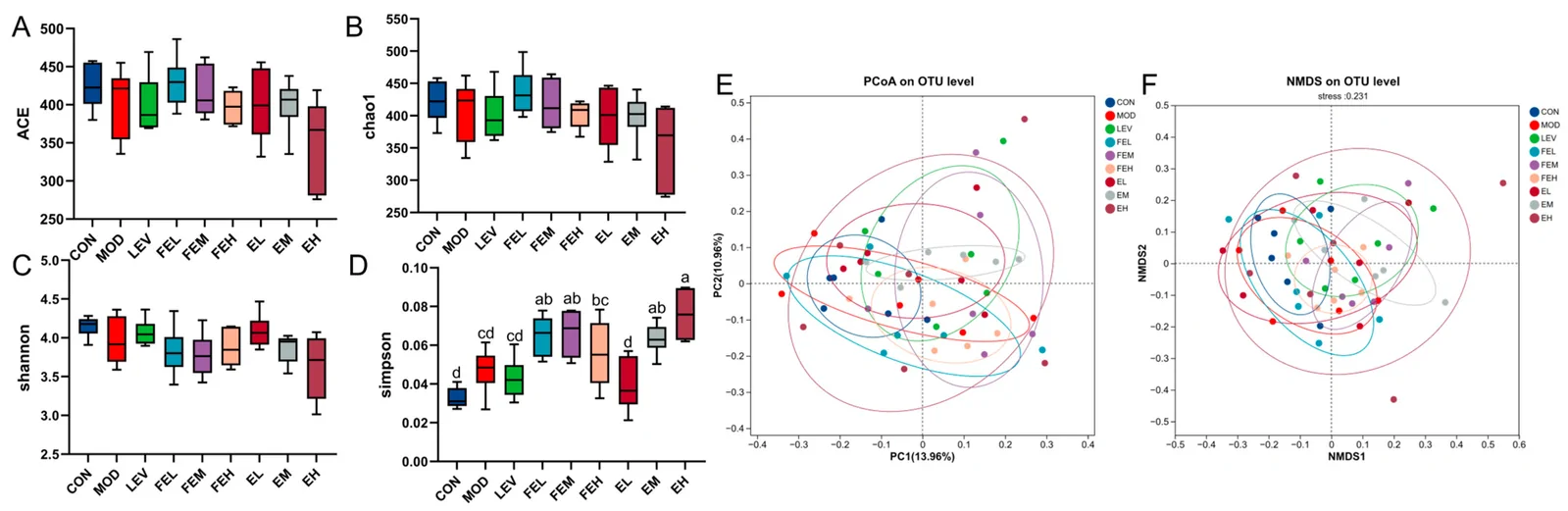
Effects of FEUPS and EUPS on the alpha and beta diversity of the mouse cecal microbiota. Alpha-diversity indices: (**A**) ACE, (**B**) Chao1, (**C**) Shannon, and (**D**) Simpson indices; beta-diversity analysis: (**E**) PCoA analysis; (**F**) NMDS analysis. Boxplots show the distribution of alpha-diversity indices across samples (*n* = 6). Different letters indicate statistically significant differences at the *p* < 0.05 level.

**Figure 8:**
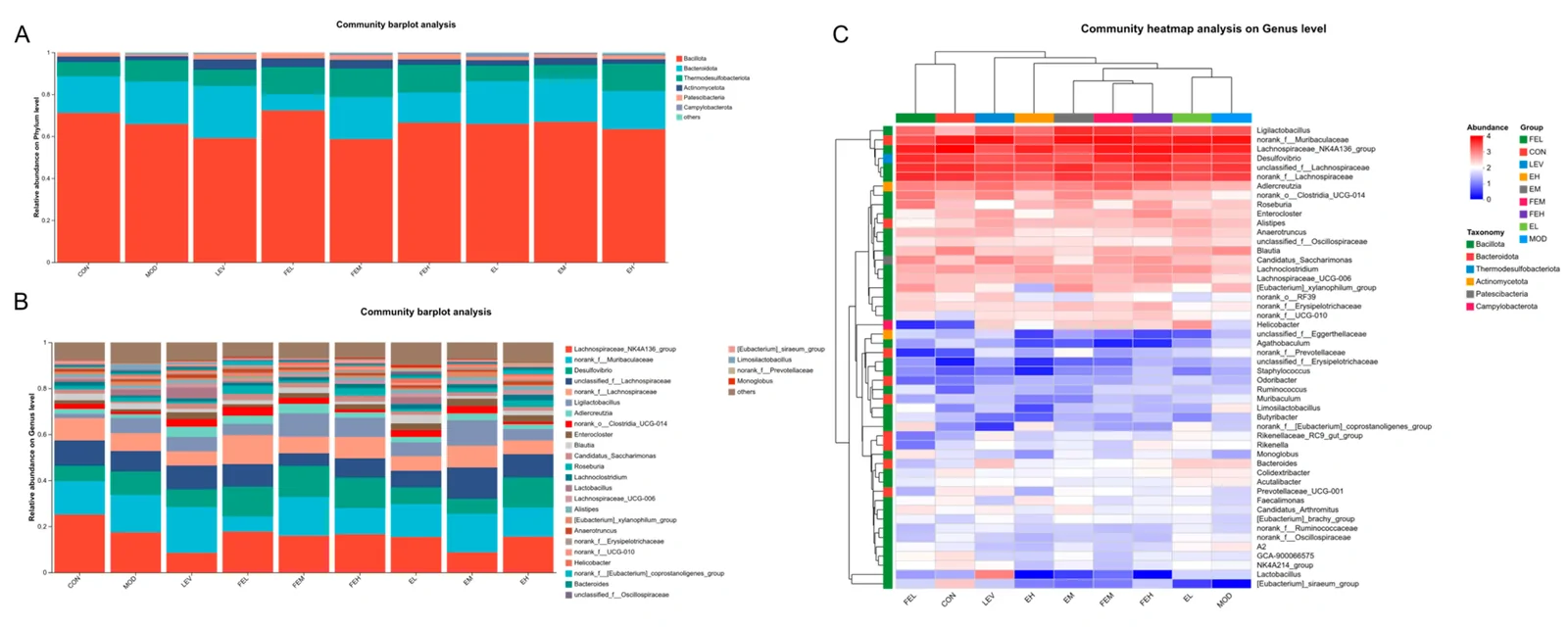
Effects of FEUPS and EUPS on the taxonomic composition of the mouse cecal microbiota. Microbial community composition at the (**A**) phylum and (**B**) genus levels; (**C**) genus-level hierarchical clustering heatmap (*n* = 6 biological replicates per group).

**Figure 9:**
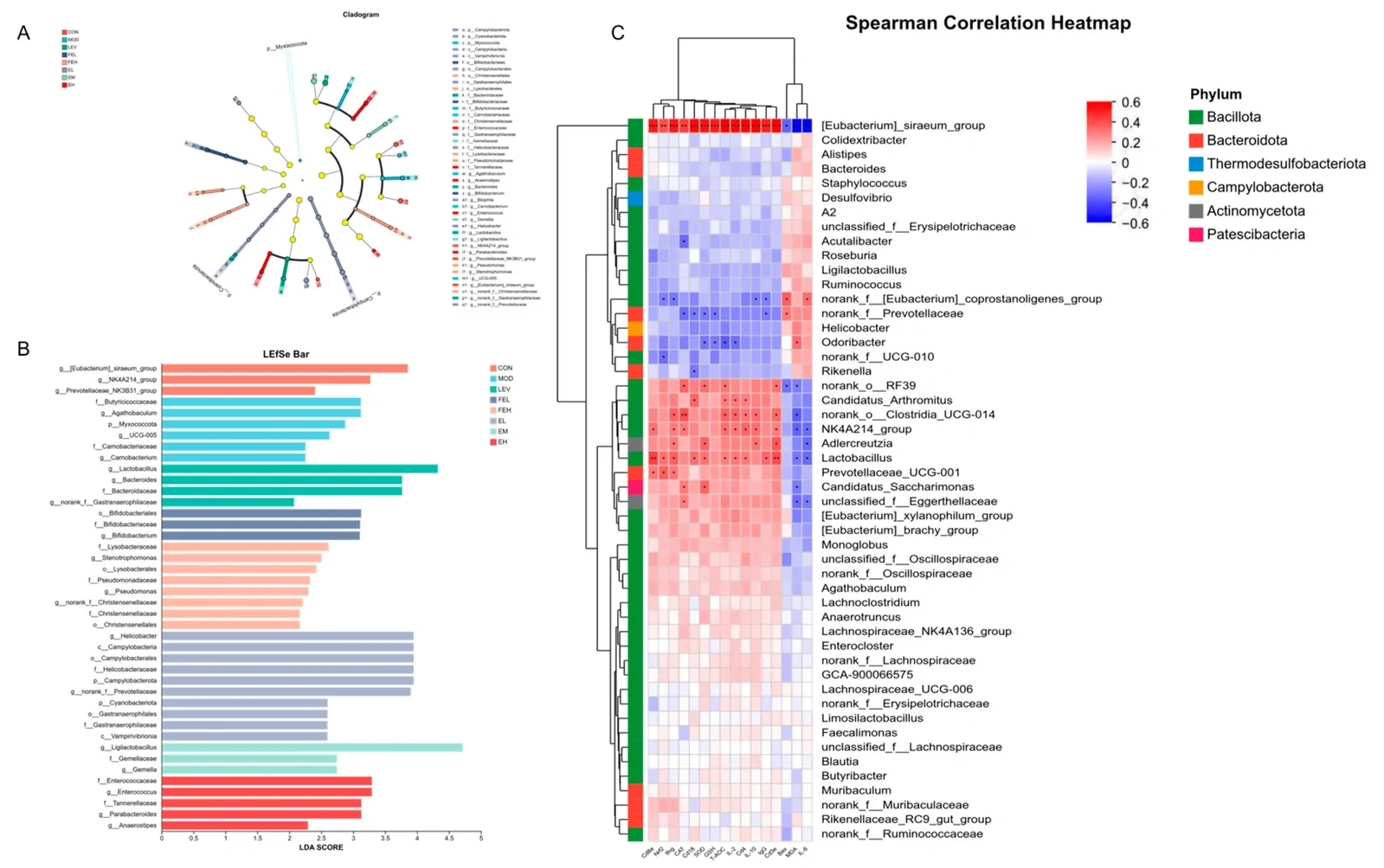
Effects of FEUPS and EUPS on the differential abundance of the mouse cecal microbiota. LEfSe analysis showing (**A**) the taxonomic cladogram and (**B**) LDA scores of differentially abundant taxa; (**C**) Spearman correlation analysis between dominant bacterial genera and host immune and antioxidant parameters (*n* = 6 biological replicates per group). *p* values were adjusted using the Benjamini–Hochberg false discovery rate (FDR) procedure. * q < 0.05, ** q < 0.01.

**Table 1:** Percentage Composition of Monosaccharides.

	FEUPS (%)	EUPS (%)
Fuc	1.35	1.50
Rha	7.72	5.05
Ara	31.14	27.91
GlcN	0.29	1.33
Gal	43.77	36.98
Xyl	2.04	2.42
Glc	7.64	17.15
Man	2.84	5.46
Gal-UA	0.54	0.78
Glc-UA	2.67	1.43

Fuc, fucose; Rha, rhamnose; Ara, arabinose; GlcN, glucosamine; Gal, galactose; Xyl, xylose; Glc, glucose; Man, mannose; Gal-UA, galacturonic acid; Glc-UA, glucuronic acid. Values represent the relative proportion of each identified monosaccharide, calculated as the concentration of the individual monosaccharide divided by the summed concentration of all identified monosaccharides. The reported values were obtained from a single HPAEC-PAD analytical determination for each sample.

**Table 2:** Organ index.

Item	Heart Index (%)	Liver Index (%)	Spleen Index (%)	Kidney Index (%)
CON	0.57 ± 0.04	4.37 ± 0.14	0.51 ± 0.05	1.53 ± 0.08
MOD	0.62 ± 0.03	4.15 ± 0.21	0.42 ± 0.03	1.48 ± 0.09
LEV	0.57 ± 0.04	4.37 ± 0.29	0.53 ± 0.07	1.51 ± 0.10
FEL	0.58 ± 0.05	4.33 ± 0.12	0.54 ± 0.06	1.50 ± 0.06
FEM	0.56 ± 0.06	4.49 ± 0.19	0.57 ± 0.04	1.50 ± 0.06
FEH	0.55 ± 0.04	4.54 ± 0.19	0.57 ± 0.07	1.53 ± 0.09
EL	0.56 ± 0.08	4.46 ± 0.30	0.56 ± 0.08	1.49 ± 0.10
EM	0.56 ± 0.07	4.37 ± 0.24	0.59 ± 0.02	1.47 ± 0.05
EH	0.58 ± 0.04	4.34 ± 0.32	0.58 ± 0.09	1.47 ± 0.08
*p*-value	0.06	0.98	0.66	0.27

The data are presented as mean ± SEM (*n* = 6 mice).

## Data Availability

The raw 16S rRNA gene sequencing data and associated sample metadata have been deposited in the NCBI Sequence Read Archive (SRA) under BioProject accession number PRJNA1529748. Other data supporting the findings of this study are available within the article and its Supplementary Materials.

## Supplementary Materials

The following supporting information can be downloaded at https://www.mdpi.com/article/10.3390/foods15193463/s1, Figure S1: Original GPC chromatograms and molecular-weight calibration profiles of EUPS and FEUPS using dextran standards; Table S1: Primer sequences; Table S2: Statistical analysis and effect sizes of the main outcome measures; Table S3: Molecular weight distributions of FEUPS and EUPS; Table S4: Relative fold-change values of spleen-related gene expression in different experimental groups; Table S5: Relative Abundance of bacterial Phyla in the cecal microbiota of different experimental groups; Table S6: Relative abundance of the top 28 bacterial genera and other genera in the cecal microbiota of different experimental groups.
