# Supplementary material for: Lactiplantibacillus plantarum Fermentation Alters the Physicochemical Characteristics and Enhances the Antioxidant and Immunomodulatory Activities of Eucommia ulmoides Leaf Polysaccharides
Source: Foods. 2026 Sep 28;15(19):3463. doi: 10.3390/foods15193463 (PMC13664025; doi:10.3390/foods15193463)
Supplement: Supplementary file 1 [file foods-15-03463-s001.zip › Figure S1. GPC chromatograms and molecular-weight calibration profiles of EUPS and FEUPS..pdf]

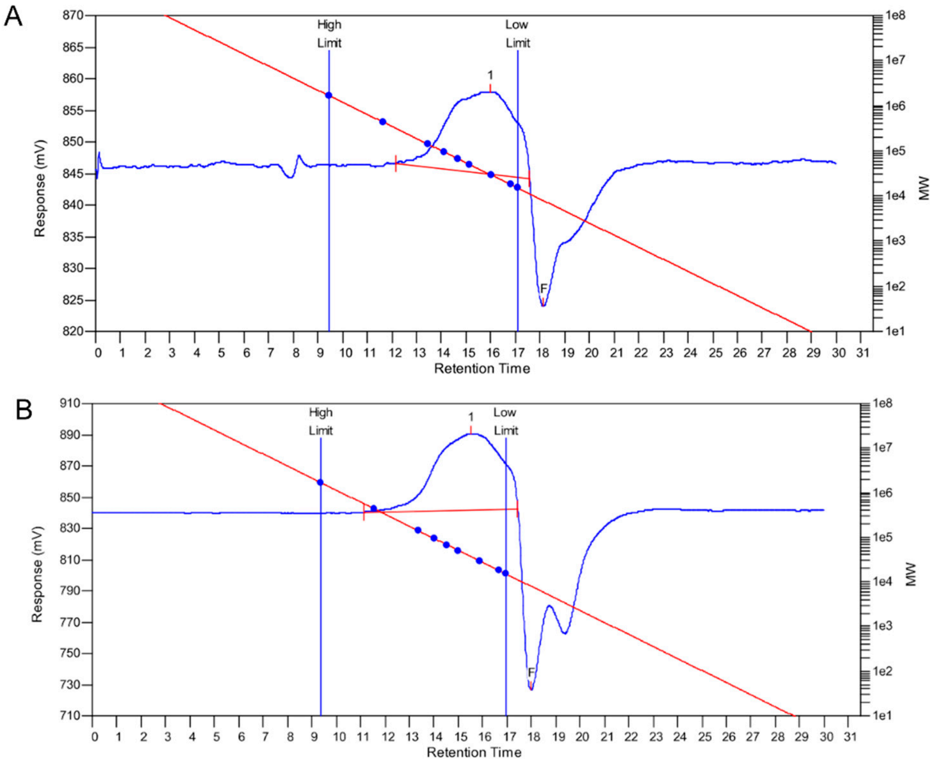

Figure S1. GPC chromatograms and molecular-weight calibration profiles of EUPS and FEUPS. (A) EUPS; (B) FEUPS.
