# Supplementary material for: Lactiplantibacillus plantarum Fermentation Alters the Physicochemical Characteristics and Enhances the Antioxidant and Immunomodulatory Activities of Eucommia ulmoides Leaf Polysaccharides
Source: Foods. 2026 Sep 28;15(19):3463. doi: 10.3390/foods15193463 (PMC13664025; doi:10.3390/foods15193463)
Supplement: Supplementary file 1 [file foods-15-03463-s001.zip › Table S1. Primer Sequences.pdf]

Table S1. Primer Sequences.

Gene	Primer sequences (5′-3′)	Serial Number	bp
IL-10	F: GGTTGCCAAGCCTTATCGGA<br>R: CACCTTGGTCTTGGAGCTTAT	NM_010548.2	226
IL-2	F: GGAACCTGAAACTCCCCAGG<br>R: TGTGTTGTCAGAGCCCTTTAGT	NM_008366.3	215
Cd3e	F: TACCTGAAAGCTCGAGTGTGTG<br>R: TAGTCTGGGTTGGGAACAGGT	NM_007648.5	227
Cd4	F: CTCCAGGATCCAGGTTTTATCC<br>R: GGGACAAGGAGAGTAGTCGTG	XM_006505462.2	208
Cd8a	F: GCCAGTCCTTCAGAAAGTGAAC<br>R: AACACGCTTTCGGCTCCTG	NM_001081110.2	250
Cd18	F: CGTGGAGGATAGTGGGGAGAT<br>R: GATTCAAACTGCTCCCCCGA	NM_001357091.2	191
Nrf2	F: GCTGCTCGGACTAGCCATTG<br>R: TGGGCAACCATCACTCTGCTG	NM_001399226.1	148
Bax	F: TCTGCTACAACGTGCTGGAT<br>R: CACCCTTCTGAATGCCCTGT	NM_019811.3	174
Ifng	F: AGGAACTGGCAAAAGGATGGT<br>R: TCATTGAATGCTTGGCGCTG	NM_008337.4	236
β-actin	F: CCTAGGCACCAGGGTGTGAT<br>R: AGCACAGGGTGCTCCTCA	NM_007393.5	200
