# Supplementary material for: Lactiplantibacillus plantarum Fermentation Alters the Physicochemical Characteristics and Enhances the Antioxidant and Immunomodulatory Activities of Eucommia ulmoides Leaf Polysaccharides
Source: Foods. 2026 Sep 28;15(19):3463. doi: 10.3390/foods15193463 (PMC13664025; doi:10.3390/foods15193463)
Supplement: Supplementary file 1 [file foods-15-03463-s001.zip › Table S2. Statistical Analysis and Effect Sizes of the Main Outcome Measures.pdf]

Table S2. Statistical Analysis and Effect Sizes of the Main Outcome Measures

	Shapiro P	Levene P	Analytical Methods	Exact P	Effect size
Change in weight	> 0.05	< 0.001	Welch's ANOVA	< 0.001	$\eta^2 = 0.539$
IgG	> 0.05	0.107	One-way ANOVA	< 0.001	$\eta^2 = 0.977$
IgM	> 0.05	0.092	One-way ANOVA	< 0.001	$\eta^2 = 0.926$
CAT	0.2644	0.490	One-way ANOVA	< 0.001	$\eta^2 = 0.844$
GSH	0.6872	0.094	One-way ANOVA	< 0.001	$\eta^2 = 0.892$
MDA	< 0.001	0.011	Kruskal–Wallis	< 0.001	$\varepsilon^2 = 0.867$
SOD	0.021	0.013	Kruskal–Wallis	< 0.001	$\varepsilon^2 = 0.875$
T-AOC	0.702	< 0.001	Welch's ANOVA	< 0.001	$\eta^2 = 0.952$
Cd3e	< 0.001	< 0.001	Kruskal–Wallis	< 0.001	$\varepsilon^2 = 0.773$
Cd4	0.073	< 0.001	Welch's ANOVA	< 0.001	$\eta^2 = 0.901$
Cd8a	0.494	< 0.001	Welch's ANOVA	< 0.001	$\eta^2 = 0.976$
Cd18	0.070	0.030	Welch's ANOVA	< 0.001	$\eta^2 = 0.972$
Bax	0.033	< 0.001	Kruskal–Wallis	< 0.001	$\varepsilon^2 = 0.599$
Nrf2	< 0.001	0.106	Kruskal–Wallis	< 0.001	$\varepsilon^2 = 0.884$
Ifng	0.232	0.080	One-way ANOVA	< 0.001	$\eta^2 = 0.951$
IL-2	0.016	0.049	Kruskal–Wallis	< 0.001	$\varepsilon^2 = 0.949$
IL-10	0.232	0.132	One-way ANOVA	< 0.001	$\eta^2 = 0.965$
IL-6	0.111	0.275	One-way ANOVA	< 0.001	$\eta^2 = 0.962$
ACE	0.450	0.208	Kruskal–Wallis	0.183	$\varepsilon^2 = 0.074$
Chao1	0.221	0.250	Kruskal–Wallis	0.285	$\varepsilon^2 = 0.038$
Shannon	0.847	0.214	Kruskal–Wallis	0.052	$\varepsilon^2 = 0.178$

Table S2. Statistical Analysis and Effect Sizes of the Main Outcome Measures

	Shapiro P	Levene P	Analytical Methods	Exact P	Effect size
Simpson	0.617	0.072	Kruskal–Wallis	< 0.001	$\varepsilon^2 = 0.570$
