# Supplementary material for: Lactiplantibacillus plantarum Fermentation Alters the Physicochemical Characteristics and Enhances the Antioxidant and Immunomodulatory Activities of Eucommia ulmoides Leaf Polysaccharides
Source: Foods. 2026 Sep 28;15(19):3463. doi: 10.3390/foods15193463 (PMC13664025; doi:10.3390/foods15193463)
Supplement: Supplementary file 1 [file foods-15-03463-s001.zip › Table S3. Molecular Weight Distributions of FEUPS and EUPS.pdf]

Table S3. Molecular Weight Distributions of FEUPS and EUPS.

	Mw（Da）	Mn（Da）	Mp(Da)	PD
FEUPS	53069	33213	37017	1.59779
EUPS	49103	31682	28023	1.54987
