# Supplementary material for: Lactiplantibacillus plantarum Fermentation Alters the Physicochemical Characteristics and Enhances the Antioxidant and Immunomodulatory Activities of Eucommia ulmoides Leaf Polysaccharides
Source: Foods. 2026 Sep 28;15(19):3463. doi: 10.3390/foods15193463 (PMC13664025; doi:10.3390/foods15193463)
Supplement: Supplementary file 1 [file foods-15-03463-s001.zip › Table S4. Relative Fold-Change Values of Spleen-Related Gene Expression in Different Experimental Groups.pdf]

Table S4. Relative Fold-Change Values of Spleen-Related Gene Expression in Different Experimental Groups

Gene	CON	MOD	LEV	FEL	FEM	FEH	EL	EM	EH
Cd3e	1.000 ± 0.146	0.154 ± 0.021	0.802 ± 0.114	0.552 ± 0.021	0.636 ± 0.061	0.318 ± 0.040	0.409 ± 0.016	0.414 ± 0.014	0.266 ± 0.042
Cd4	1.000 ± 0.056	0.312 ± 0.022	0.846 ± 0.011	0.764 ± 0.015	0.816 ± 0.019	0.534 ± 0.007	0.637 ± 0.039	0.678 ± 0.038	0.465 ± 0.025
Cd8a	1.000 ± 0.013	0.153 ± 0.012	0.688 ± 0.011	0.560 ± 0.029	0.625 ± 0.016	0.423 ± 0.009	0.458 ± 0.008	0.522 ± 0.020	0.330 ± 0.013
Cd18	1.000 ± 0.026	0.225 ± 0.024	0.817 ± 0.010	0.523 ± 0.008	0.637 ± 0.023	0.434 ± 0.015	0.469 ± 0.009	0.506 ± 0.008	0.407 ± 0.014
Bax	1.000 ± 0.007	1.735 ± 0.095	1.255 ± 0.007	1.355 ± 0.077	1.276 ± 0.012	1.382 ± 0.059	1.444 ± 0.105	1.414 ± 0.019	1.526 ± 0.070
Nrf2	1.000 ± 0.054	0.322 ± 0.030	0.822 ± 0.010	0.540 ± 0.020	0.723 ± 0.020	0.388 ± 0.034	0.455 ± 0.044	0.530 ± 0.015	0.357 ± 0.013
Ifng	1.000 ± 0.019	0.213 ± 0.028	0.819 ± 0.025	0.535 ± 0.023	0.770 ± 0.010	0.363 ± 0.021	0.374 ± 0.014	0.471 ± 0.045	0.317 ± 0.028
IL-2	1.000 ± 0.025	0.211 ± 0.028	0.767 ± 0.039	0.561 ± 0.026	0.715 ± 0.007	0.429 ± 0.022	0.470 ± 0.012	0.525 ± 0.008	0.339 ± 0.011
IL-10	1.000 ± 0.025	0.237 ± 0.018	0.791 ± 0.033	0.495 ± 0.022	0.735 ± 0.020	0.315 ± 0.009	0.450 ± 0.017	0.535 ± 0.012	0.255 ± 0.023
IL-6	1.000 ± 0.021	2.535 ± 0.044	1.427 ± 0.029	1.745 ± 0.016	1.550 ± 0.013	1.926 ± 0.050	1.815 ± 0.053	1.753 ± 0.030	2.063 ± 0.041

Data are presented as mean ± SEM (n = 6). Relative gene expression values were normalized to the mean value of the corresponding CON group, which was set to 1.000.
