# Supplementary material for: Lactiplantibacillus plantarum Fermentation Alters the Physicochemical Characteristics and Enhances the Antioxidant and Immunomodulatory Activities of Eucommia ulmoides Leaf Polysaccharides
Source: Foods. 2026 Sep 28;15(19):3463. doi: 10.3390/foods15193463 (PMC13664025; doi:10.3390/foods15193463)
Supplement: Supplementary file 1 [file foods-15-03463-s001.zip › Table S5. Relative Abundance of Bacterial Phyla in the Cecal Microbiota of Different Experimental Groups.pdf]

Table S5. Relative Abundance of Bacterial Phyla in the Cecal Microbiota of Different Experimental Groups

Phylum	CON	MOD	LEV	FEL	FEM	FEH	EL	EM	EH
Actinomycetota	2.63 ± 0.63	1.98 ± 0.92	4.89 ± 1.33	4.11 ± 1.49	4.30 ± 0.81	2.46 ± 0.71	2.53 ± 0.97	3.40 ± 1.05	2.13 ± 0.61
Bacillota	71.21 ± 4.37	65.82 ± 5.02	59.10 ± 8.18	72.32 ± 4.89	58.64 ± 3.99	66.53 ± 3.03	65.86 ± 3.62	66.83 ± 5.61	63.44 ± 8.73
Bacteroidota	17.38 ± 3.53	20.27 ± 3.89	24.88 ± 7.40	7.81 ± 2.30	20.02 ± 4.34	14.35 ± 1.74	20.43 ± 3.86	20.63 ± 3.68	18.16 ± 8.68
Campylobacterota	0.03 ± 0.01	0.63 ± 0.45	0.68 ± 0.08	0.01 ± 0.01	1.16 ± 0.49	0.65 ± 0.15	1.85 ± 0.41	0.80 ± 0.14	0.89 ± 0.37
Cyanobacteriota	0.01 ± 0.01	0.01 ± 0.00	0.06 ± 0.03	0.01 ± 0.01	0.02 ± 0.01	0.00 ± 0.00	0.07 ± 0.04	0.01 ± 0.00	0.05 ± 0.05
Deferribacterota	0.02 ± 0.00	0.03 ± 0.02	0.01 ± 0.00	0.05 ± 0.03	0.04 ± 0.02	0.03 ± 0.01	0.09 ± 0.04	0.02 ± 0.01	0.04 ± 0.02
Myxococcota	0.00 ± 0.00	0.00 ± 0.00	0.00 ± 0.00	0.00 ± 0.00	0.00 ± 0.00	0.00 ± 0.00	0.00 ± 0.00	0.00 ± 0.00	0.00 ± 0.00
Patescibacteria	1.85 ± 0.92	0.74 ± 0.24	2.50 ± 0.56	2.73 ± 0.76	2.12 ± 0.63	2.60 ± 1.16	1.59 ± 0.65	1.65 ± 0.68	1.75 ± 0.68
Pseudomonadota	0.03 ± 0.01	0.30 ± 0.17	0.08 ± 0.04	0.03 ± 0.01	0.10 ± 0.04	0.08 ± 0.04	0.11 ± 0.03	0.09 ± 0.04	0.50 ± 0.47
Thermodesulfobacteriota	6.84 ± 1.12	10.22 ± 3.16	7.80 ± 3.06	12.92 ± 3.97	13.59 ± 3.57	13.31 ± 2.58	7.47 ± 1.59	6.56 ± 2.24	13.04 ± 6.64
unclassified_k__norank_d__Bacteria	0.00 ± 0.00	0.01 ± 0.00	0.00 ± 0.00	0.00 ± 0.00	0.00 ± 0.00	0.00 ± 0.00	0.00 ± 0.00	0.00 ± 0.00	0.01 ± 0.01

Data are presented as mean ± SEM (n = 6) and expressed as relative abundance (%).
