# Supplementary material for: Lactiplantibacillus plantarum Fermentation Alters the Physicochemical Characteristics and Enhances the Antioxidant and Immunomodulatory Activities of Eucommia ulmoides Leaf Polysaccharides
Source: Foods. 2026 Sep 28;15(19):3463. doi: 10.3390/foods15193463 (PMC13664025; doi:10.3390/foods15193463)
Supplement: Supplementary file 1 [file foods-15-03463-s001.zip › Table S6. Relative Abundance of the Top 28 Bacterial Genera.pdf]

Table S6. Relative Abundance of the Top 28 Bacterial Genera and Other Genera in the Cecal Microbiota of Different Experimental Groups

Genus	CON	MOD	LEV	FEL	FEM	FEH	EL	EM	EH
Lachnospiraceae_NK4A136_group	25.14 ± 3.09	17.39 ± 6.00	8.48 ± 3.74	17.71 ± 5.44	15.88 ± 4.40	16.57 ± 3.43	15.35 ± 2.24	8.72 ± 2.56	15.56 ± 5.32
norank_f__Muribaculaceae	14.46 ± 3.04	16.31 ± 2.91	19.91 ± 6.18	6.64 ± 1.99	16.90 ± 3.64	11.36 ± 1.32	14.32 ± 2.33	16.79 ± 3.11	12.73 ± 6.32
Desulfovibrio	6.81 ± 1.12	10.20 ± 3.16	7.73 ± 3.06	12.90 ± 3.97	13.52 ± 3.59	13.28 ± 2.59	7.33 ± 1.59	6.50 ± 2.22	12.98 ± 6.66
unclassified_f__Lachnospiraceae	10.93 ± 2.18	8.89 ± 1.23	10.38 ± 3.36	9.79 ± 1.54	5.44 ± 0.52	8.37 ± 1.59	7.22 ± 1.17	13.60 ± 5.61	10.15 ± 2.46
norank_f__Lachnospiraceae	9.63 ± 1.76	7.69 ± 1.43	6.10 ± 0.99	12.62 ± 4.02	7.26 ± 1.58	9.22 ± 0.75	6.26 ± 1.27	9.44 ± 1.88	5.86 ± 1.78
Ligilactobacillus	1.90 ± 0.84	6.62 ± 1.85	6.30 ± 2.78	4.92 ± 1.64	10.12 ± 2.43	8.42 ± 2.39	6.05 ± 2.29	11.13 ± 2.47	5.02 ± 2.03
Adlercreutzia	2.24 ± 0.52	1.68 ± 0.77	4.47 ± 1.24	3.57 ± 1.47	4.20 ± 0.78	2.38 ± 0.68	2.40 ± 0.95	2.92 ± 0.84	2.06 ± 0.58
norank_o__Clostridia_UCG-014	2.08 ± 0.78	1.02 ± 0.52	3.27 ± 0.96	3.76 ± 1.27	2.54 ± 1.12	1.25 ± 0.49	2.87 ± 1.39	3.18 ± 0.80	0.94 ± 0.33
Enterocloster	1.63 ± 0.74	1.13 ± 0.54	2.99 ± 1.48	1.18 ± 0.76	2.07 ± 0.96	2.18 ± 0.41	2.90 ± 1.87	3.24 ± 1.91	3.00 ± 1.47
Blautia	2.90 ± 0.82	2.59 ± 0.74	1.41 ± 0.62	1.81 ± 1.21	2.47 ± 1.42	1.20 ± 0.33	3.78 ± 1.93	1.18 ± 0.33	1.95 ± 1.05
Candidatus_Saccharimonas	1.85 ± 0.92	0.73 ± 0.23	2.50 ± 0.56	2.73 ± 0.76	2.12 ± 0.62	2.60 ± 1.17	1.59 ± 0.65	1.65 ± 0.68	1.75 ± 0.68
Roseburia	1.44 ± 0.65	1.81 ± 0.75	0.81 ± 0.47	3.44 ± 1.11	0.86 ± 0.51	3.25 ± 1.94	1.05 ± 0.42	1.80 ± 0.53	2.73 ± 1.55

Lachnoclostridium	1.86 ± 0.48	0.82 ± 0.12	1.24 ± 0.35	1.33 ± 0.36	1.45 ± 0.47	1.84 ± 0.46	1.99 ± 0.49	1.69 ± 0.51	2.52 ± 1.28
Lactobacillus	1.04 ± 0.99	2.56 ± 1.29	4.80 ± 1.82	0.78 ± 0.66	0.29 ± 0.25	0.00 ± 0.00	3.03 ± 2.44	0.03 ± 0.02	0.00 ± 0.00
Lachnospiraceae_UCG-006	0.93 ± 0.28	0.52 ± 0.14	1.85 ± 1.06	1.22 ± 0.34	1.01 ± 0.24	1.49 ± 0.23	1.67 ± 0.87	1.70 ± 0.52	2.00 ± 0.90
Alistipes	1.25 ± 0.77	1.29 ± 0.47	1.48 ± 0.32	0.62 ± 0.33	0.87 ± 0.17	1.43 ± 0.49	1.66 ± 0.43	1.38 ± 0.39	1.73 ± 1.05
[Eubacterium]_xylanophilum_group	1.07 ± 0.25	1.68 ± 1.03	0.86 ± 0.28	1.84 ± 0.39	1.11 ± 0.27	0.80 ± 0.16	0.67 ± 0.27	2.24 ± 0.67	0.59 ± 0.37
Anaerotruncus	1.31 ± 0.44	0.95 ± 0.27	1.46 ± 0.45	1.50 ± 0.40	0.81 ± 0.34	1.02 ± 0.34	1.12 ± 0.25	0.85 ± 0.26	1.46 ± 0.63
norank_f__Erysipelotrichaceae	0.51 ± 0.08	0.59 ± 0.22	0.65 ± 0.19	0.95 ± 0.31	0.65 ± 0.07	1.35 ± 0.51	0.30 ± 0.07	1.17 ± 0.45	0.76 ± 0.25
norank_f__UCG-010	0.16 ± 0.04	0.78 ± 0.35	0.53 ± 0.10	0.69 ± 0.20	0.65 ± 0.21	1.16 ± 0.36	0.79 ± 0.46	0.61 ± 0.21	1.55 ± 0.77
Helicobacter	0.03 ± 0.01	0.63 ± 0.45	0.68 ± 0.08	0.01 ± 0.01	1.16 ± 0.49	0.65 ± 0.15	1.85 ± 0.41	0.80 ± 0.14	0.89 ± 0.37
norank_f__[Eubacterium]_coprostanoligenes_group	0.29 ± 0.26	0.55 ± 0.37	0.07 ± 0.07	1.68 ± 0.68	0.18 ± 0.08	0.37 ± 0.21	0.87 ± 0.38	0.17 ± 0.06	1.58 ± 0.79
Bacteroides	0.34 ± 0.17	0.92 ± 0.35	1.10 ± 0.43	0.13 ± 0.05	0.37 ± 0.17	0.34 ± 0.06	0.90 ± 0.30	0.30 ± 0.12	1.01 ± 0.88
unclassified_f__Oscillospiraceae	0.66 ± 0.08	0.61 ± 0.19	0.57 ± 0.13	0.43 ± 0.06	0.52 ± 0.18	0.38 ± 0.08	1.20 ± 0.66	0.51 ± 0.10	0.47 ± 0.08
[Eubacterium]_siraeum_group	1.42 ± 0.56	0.00 ± 0.00	0.83 ± 0.77	0.45 ± 0.34	0.12 ± 0.10	1.37 ± 1.24	0.03 ± 0.02	0.27 ± 0.25	0.77 ± 0.74

Limosilactobacillus	0.17 ± 0.15	2.47 ± 1.30	0.76 ± 0.48	0.47 ± 0.25	0.20 ± 0.08	0.27 ± 0.12	0.26 ± 0.22	0.16 ± 0.07	0.01 ± 0.01
Prevotellaceae_UCG-001	0.56 ± 0.17	0.28 ± 0.12	0.74 ± 0.42	0.13 ± 0.08	0.64 ± 0.36	0.19 ± 0.04	0.68 ± 0.35	0.85 ± 0.42	0.61 ± 0.51
norank_o__RF39	0.41 ± 0.10	0.37 ± 0.14	0.81 ± 0.20	0.64 ± 0.12	0.56 ± 0.18	0.85 ± 0.42	0.37 ± 0.22	0.30 ± 0.14	0.24 ± 0.08
Others	6.97 ± 0.70	8.92 ± 0.93	7.23 ± 0.86	6.07 ± 0.44	6.04 ± 1.20	6.41 ± 0.39	11.47 ± 2.10	6.83 ± 0.78	9.08 ± 2.16

Data are presented as mean ± SEM (n = 6) and expressed as relative abundance (%). Others represents all genera outside the top 28.
